# 

**Published:** 1792

**Authors:** 


### CONTENTS.

Page

I. *CASES of Iſchuria Renalis in Children. By* Robert Willan, *M. D. F. A. S. Phyſician to the Public Diſpenſary in London.* — — 1

II. *A Caſe of Pemphigus. By* T. M. Winterbottom, *M. D. Phyſician to the Settlement at Sierra Leone.* — 10

III. *Caſe of Injury of the Brain, without a Fracture, relieved by Application of the Trephine. By Mr.* John Andrews, *Surgeon in London.* — — 12

IV. *Caſe of a Cyſt containing Hydatids, extracted from the right anterior Ventricle of the Brain of a Cow. By Mr.* William Moorcroft, *Veterinarian Surgeon in London.* — — 17

V. *Facts relative to the Prevention of Hydrophobia. By Mr.* Jeſſe Foot, *Surgeon in London.* — —— 33

VI. *Two Caſes of Fracture; one of the upper, the other of the lower Jaw. By Mr.* T. Hughes,

Page

Hughes, *Surgeon at Stroud-Water in Gloucesterſhire.* — — 36

VII. *Caſe of an enlarged Nympha. By Mr.* William Morlen, *Surgeon in London.* 50

VIII. *An Account of the good Effects of Electricity in a Caſe of violent ſpaſmodic Affection. By Mr.* George Wilkinſon, *Surgeon at Sunderland, and Member of the Royal College of Surgeons at Edinburgh, &c.* — — — 52

IX. *Caſe of a ſingular cutaneous Affection; with ſome Remarks relative to the Poiſon of Copper. By Mr.* William Davidſon, *Apothecary in London.* — — 61

X. *Two Caſes of pulmonary Hæmorrhage, ſpeedily and ſucceſsfully cured by Abſtinence from Liquids. By the Same.* — 68

XI. *An Account of a Diſeaſe which, until lately, proved fatal to a great Number of Infants in the Lying-in Hoſpital of Dublin; with Obſervations on its Cauſes and Prevention. By* Joſeph Clarke, *M. D. Maſter of the Hoſpital above mentioned, and M. R. I. A. From the Tranſactions of the Royal Iriſh Academy.* — 78

XII. *Obſervations on certain horny Excreſcences of the human Body. By* Everard Home,

Page

Home, *Esq. F. R. S. From the Philosophical Transactions of the Royal Society of London.* — — 105

XIII. *Experiments on Human Calculi. In a Letter from Mr.* Timothy Lane, *F. R. S. to* William Pitcairn, *M. D. F. R. S. From t e same W rk.* — 121

XIV. *Experiments and Observations to investigate the Composition f James's Powder. By* George Pearson, *M. D. F. R. S. From the same Work.* —— 128

XV. *Account of a Case of double Hare Lip, accompanied with a Fissure of the Palate; with Remarks. By M.* Chorin, *one of the Surgeons of the Hotel Dieu at Paris. From the Journal de Chirurgie.* 153

XVI. *An Account of a Child who drinks a great Quantity of Water. By M.* Vauquelin. *From a Work entitled La Medecine Eclairée par les Sciences physiques, ou Journal des Découvertes relatives aux differentes Parties de l'Art de Guerir.* 167

XVII. *A Case of double Uterus. By* Antonio Canestrini, *Physician to the Imperial Mines at Schwatz in Tyrol. Translated from the German.* — — 171

XVIII. *An*

Page

XVIII. *An Account of the Experiments and Difcoveries of* Lewis Galvani, *Profeffor of Anatomy at Bologna, relative to the Powers of Electricity in Mufcular Motion.* 180

XIX. *Two Letters on Animal Electricity. By* Eufebius Valli, *M. D. of the Univerfity of Pifa. From the Journal de Phyfique.* 190

XX. *Additional Obfervations on Animal Electricity. By* Eufebius Valli, *M. D.* 212

*Catalogue of Books.* — — 218

*Index.* — — — 227

